# IZUMO1 Receptor Localization during Hyaluronic Acid Selection in Human Spermatozoa

**DOI:** 10.3390/biomedicines11112872

**Published:** 2023-10-24

**Authors:** María José Gómez-Torres, Miranda Hernández-Falcó, Andrea López-Botella, Natalia Huerta-Retamal, Paula Sáez-Espinosa

**Affiliations:** 1 Department of Biotechnology, University of Alicante, 03690 Alicante, Spain; mjose.gomez@ua.es (M.J.G.-T.); mhf7@alu.ua.es (M.H.-F.);; 2 Human Fertility Cathedra, University of Alicante, 03690 Alicante, Spain

**Keywords:** acrosome reaction, gamete fusion, IZUMO1, sperm selection, male infertility, immunofluorescent, hyaluronic acid, sperm capacitation

## Abstract

IZUMO1 is an acrosome transmembrane protein implicated in the adhesion and fusion of gametes. This study aims to describe the distribution of IZUMO1 in human sperm under different physiological conditions: before capacitation (NCS), at one-hour capacitation (CS1), after a hyaluronic acid (HA) selection test (mature, MS1 and immature, IS1), and induced acrosome reaction from one-hour-capacitated sperm (ARS1). The data obtained in NCS, CS1, and MS1 significantly highlight dotted fluorescence in the acrosomal region (P1) as the major staining pattern (~70%). Moreover, we describe a new distribution pattern (P2) with a dotted acrosomal region and a labelled equatorial region that significantly increases in HA-bound spermatozoa, suggesting the onset of the migration of IZUMO1. In contrast, unbound spermatozoa presented an increase in P3 (equatorial region labelled) and P4 (not labelled). Finally, costaining to observe IZUMO1 distribution and acrosome status was performed in ARS1. Interestingly, we reported a variety of combinations between the IZUMO1 staining patterns and the acrosomal stages. In conclusion, these data show as a novelty the diffusion of the IZUMO1 protein during different physiological conditions that could contribute to the improvement in sperm selection techniques.

## 1. Introduction

The capacity of spermatozoa to fertilize is acquired during their ascent through the female tract. This time-dependent process, known as capacitation, involves a set of structural and physiological changes including, among others, cholesterol loss [1], reorganization of glycoconjugates [2], and protein phosphorylation [3], in addition to developing the potential to disperse the cumulus–oophorus complex (COC) surrounding the oocyte [4]. As a result of capacitation, spermatozoa acquire the ability to undergo acrosomal reaction. This highly regulated exocytotic event allows the spermatozoa to penetrate the zona pellucida, the sperm–oocyte binding, and the gamete fusion. All these events are essential to achieve successful fertilization [5,6].

Due to its role as a physiological sorter, the extracellular matrix of COC has been an important focus of research in recent decades. This complex structure is mainly composed of hyaluronic acid (HA) produced by the cumulus cells [7]. It has been documented that only the spermatozoa that have undergone cellular maturation, including plasma membrane remodeling, can bind to and digest HA [8,9], showing better motility, morphological characteristics, and nuclear maturity, as well as the lower DNA fragmentation levels, lower risk of chromosomal imbalance, and presence of an intact acrosome [10,11,12]. These spermatozoa were termed by Cayli et al. [13] as “mature” spermatozoa, while those with deficient maturity and plasma membrane remodeling were named “immature”.

Based on the statement that the presence of HA receptors reflects their maturity [14], the HA selection method has been extensively studied as a potential technique for improving fertility outcomes in assisted reproductive technologies (ARTs). In particular, HA-based sperm selection using a solid-state physiological ICSI (PICSI) compared to ICSI, showed promising results in reducing miscarriage rates even though there were no statistically significant differences in live births [15]. In addition, recent research has reported increased fertilization and embryo development rates [12,14,16,17,18]. Despite the outcomes observed, the existing controversy about the suitability of this technique compared to other current treatments emphasizes the need for further research [15].

After physiological selection by COC, mature spermatozoa must be able to recognize and adhere to oocyte receptors to achieve gamete fusion. During the last decade, only a few acrosomal proteins have been identified as fertilization-critical proteins, targeting IZUMO1 as a key adhesion and fusion protein [19].

IZUMO1 is a single-pass type I transmembrane protein located at the outer acrosomal membrane in acrosome-intact spermatozoa [20]. In mice, it has been observed that during the acrosome reaction, IZUMO1 gradually moves toward the equatorial segment, assisted by the actin cytoskeleton and testis-specific serine kinase (TSSK6) [21,22] to be able to bind its oocyte receptor named JUNO [23,24,25]. In addition, Sosnik et al. reported that TSSK6 is involved in IZUMO1 redistribution through the regulation of actin polymerization after the acrosomal reaction. Indeed, the involvement of F-actin via histone acetylation regulation has recently been demonstrated, being located at the apical part of the midpiece [26].

The role of IZUMO1 during sperm–oocyte fusion is evidenced when JUNO rearranges IZUMO1 to provide the necessary strength to break the repulsion between the juxtaposed membranes [27]. Spermatozoa lacking IZUMO1 can penetrate the zona pellucida; however, they are unable to fuse with the oocyte, accumulating in the perivitelline space [28]. In this way, the study of IZUMO1 location using the ability of HA to selectively bind to mature sperm may provide insight into the molecular mechanisms underlying successful mammalian fertilization. Understanding these mechanisms is critical for advancing the field of reproductive biology and may ultimately lead to new treatments for infertility.

Hence, this study aims to characterize in detail the location of the IZUMO1 protein in human sperm. Specifically, we analyzed the location of this protein before sperm capacitation, at one-hour capacitation, and after the HA selection test. Additionally, we coevaluated the location of IZUMO1 acrosome status after acrosome reaction induction with calcium ionophore A23187.

## 2. Materials and Methods

### 2.1. Experimental Design

**Figure 1:**
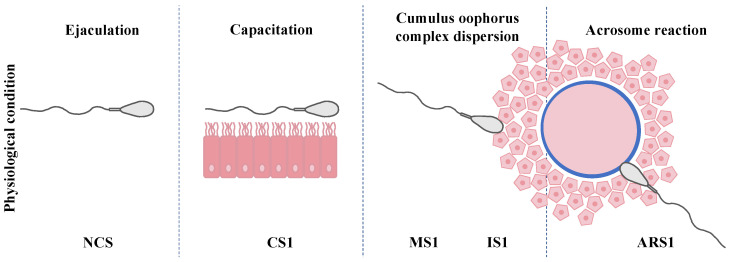
Schematic description of the experimental design used to select the different physiological conditions in this study. NCS, noncapacitated sperm; CS1, one-hour-capacitated sperm; MS1 and IS1, mature and immature sperm selected by hyaluronic acid after one-hour capacitation; and ARS1, induced acrosome reaction from one-hour-capacitated sperm.

Based on previous studies from our group [29,30], the semen samples were processed to obtain spermatozoa under the following conditions (Figure 1): noncapacitated sperm (NCS), one-hour-capacitated sperm (CS1), mature and immature sperm selected by HA after one-hour capacitation (MS1 and IS1), and induced acrosome reaction from one-hour-capacitated sperm (ARS1). Mature and immature groups were termed by Cayli et al. [13]. Mature sperm (MS) represent sperm cells that have achieved cellular maturity, expressing hyaluronic acid receptors that allow the cells to bind hyaluronic acid and expose the receptors necessary to carry out the fertilization process through sperm–oocyte binding and gamete fusion. In contrast, immature cells are those unbound sperm that have not reached cellular maturity. IZUMO1 protein and acrosome status were evaluated in all experimental conditions.

### 2.2. Semen Sample Analysis

Semen samples were obtained from 10 healthy donors aged 20–30 years by masturbation after three to four days of sexual abstinence. Informed consent was obtained from each donor. Basic semen analysis was performed within one hour of sample collection. Sperm concentration and motility were assessed using a Makler counting chamber (BioCare Europe, Rome, Italy); morphology was analyzed using Papanicolaou staining (Panreac Química S.L.U., Barcelona, Spain); and viability was studied using Sperm VitalStain^TM^ (NidaCon International AB, Mölndal, Sweden).

### 2.3. Sperm Capacitation by Swim-Up

The process used for sperm capacitation was previously described by our group [2]. Seminal plasma was therefore first removed by centrifugation for 10 min at 300× *g,* and the pellet was washed with human tubal fluid medium (HTF, Origio^®^, Måløv, Denmark). Then, spermatozoa were incubated with HTF medium supplemented with 5 mg/mL of bovine serum albumin (BSA, Sigma-Aldrich^®^, St. Louis, MO, USA) at 37 °C with 5.5% (*v*/*v*) of CO_2_ for 1 h. Next, the supernatant fraction was collected and rinsed three times in sterile-filtered Dulbecco’s phosphate-buffered saline without calcium, magnesium, and phenol red (Capricorn Scientific GmbH, Ebsdorfergrund, Germany) by centrifugation (250× *g*, 10 min). Following the capacitation, the concentration and motility were analyzed. At this point, the recovered motile sperm were divided into four aliquots: one for the HA test, another for the induction of the acrosome reaction, another for the study of the acrosomal state, and the last for fixation assigned to the analysis of IZUMO1.

### 2.4. Hyaluronic Acid Sperm Selection

A 15 μL drop of CS1 was connected with a pipette tip to a 15 μL drop of SpermSlow medium (Origio^®^, Måløv, Denmark) in a Petri dish. After 10 min of incubation at 37 °C under oil (FertiCult^TM^ Mineral Oil, FertilPro, Beemen, Belgium), the sperm with HA receptors were trapped in the junction area of the two drops and received the name mature sperm (MS1), whereas sperm without these receptors were able to swim through the SpermSlow medium droplet and were termed immature (IS1). This methodology was performed following previous protocols of our group [29,30].

### 2.5. Induction and Evaluation of Acrosomal Reaction

The induction of the acrosome reaction was performed by adding 10 μM of calcium ionophore A23187 (Sigma-Aldrich^®^, St. Louis, MO, USA) and 2 mM of calcium chloride (Panreac Química S.L.U, Barcelona, Spain) at 37 °C with 5.5% (*v*/*v*) of CO_2_, for 1 h following previous protocols of our group [2]. Only calcium chloride was added to the control cells.

The acrosome reaction induction was verified by fixing 5 μL of sample on coverslips with methanol for 30 min. After three washes with PBS, the samples were incubated with *Pisum sativum agglutinin* lectin conjugated with fluorescein-5-isothiocyanate (PSA-FITC, Sigma-Aldrich^®^, St. Louis, MO, USA) at a final concentration of 50 μg/mL for 30 min and washed three times with PBS. Finally, the samples were mounted using Fluoroshield^TM^ with 4′,6-diamidine-2′-phenylindole dihydrochloride (Sigma-Aldrich^®^, St. Louis, MO, USA) [31].

### 2.6. Fixation

All sperm physiological conditions (NCS, CS1, MS1, IS1, and ARS1) were fixed in 2% (*w*/*v*) paraformaldehyde (Electron Microscopy Sciences, Hatfield, PA, USA) diluted in PBS for 45 min at 4 °C. Afterward, the fixative solution was replaced with PBS to reach a final concentration of 10 mill/mL and the samples were stored at 4 °C until their use.

### 2.7. Immunolocation of IZUMO1

A total of 5 μL of each paraformaldehyde-fixed sample was placed on a coverslip. When the smear was dry, cells were washed twice with PBS for 5 min. Then, the smears were permeabilized with 5% (*w*/*v*) Triton X-100 for 10 min and blocked for 15 min in 2% (*w*/*v*) BSA–PBS. Afterward, smears were incubated with anti-IZUMO1 antibody (1:100) produced in rabbit (Biorbyt Ltd., Cambridge, United Kingdom) overnight at 4 °C. Subsequently, cells were rinsed and incubated for 1 h at room temperature in darkness with a secondary anti-rabbit antibody conjugated with Cy^3^ (1:100, Jackson ImmmunoResearch, Ely, United Kingdom). Finally, the cells were rinsed and mounted using Fluoroshield^TM^ with DAPI. As a negative control, the primary antibody was omitted from the experiments.

### 2.8. Immunofluorescent Costaining of IZUMO1 and Acrosome Status

The costaining with multiple antibodies allows the examination of the codistribution of two (or more) different antigens in the same cell and determines relationships between them. Therefore, to evaluate the IZUMO1 localization in the ARS1 condition, the smear was triple-stained with anti-IZUMO1 antibody, PSA–FITC, and DAPI.

### 2.9. Statistical Analysis

A minimum of 200 cells were evaluated in each condition using a Confocal Laser Scanning Zeiss LSM 800 Microscope (Zeiss, Oberkochen, Germany) with an oil 100× objective and using 405 nm, 488 nm, and 561 nm lasers. Additionally, the use of appropriate negative controls demonstrated the specificity of the reagents. It should be noted that the negative controls for IZUMO1 were performed without the first antibody, as well as the lectin omitted from the acrosomal reaction. In ARS1, the acrosome status was first determined from each cell and afterward its distribution pattern of IZUMO1 was identified. The IZUMO1 staining patterns in the sperm head and acrosome status were quantified as percentages (%).

As a result of the normal distribution of the data (Shapiro–Wilk test; *p* > 0.05), statistical differences between groups were examined using two-way analysis of variance (ANOVA), followed by univariate analysis and Bonferroni post hoc tests. Descriptive (mean ± standard deviation; SD) and statistical procedures were performed using IBM SPSS Statistics 28.0 (IBM, Armonk, NY, USA). Statistical significance was defined as a two-sided *p*-value ≤ 0.05.

## 3. Results

### 3.1. Seminal Sample Analysis

After the seminal analysis of the samples, the following results (mean ± SD) were obtained: a concentration of 121.50 ± 50.02 × 10^6^ cells/mL with 78.27 ± 9.68% of progressive motility, 9.81 ± 2.25% of normal morphology, and viability of 91.38 ± 4.94%. After 1 h capacitation, the samples showed a concentration of 40.19 ± 21.91 × 10^6^ cells/mL with 94.07 ± 3.81% of progressive motility. According to the WHO guidelines [32], all semen samples were classified as normozoospermic.

### 3.2. Acrosome Reaction Assesment

The acrosome status was classified into three different staining patterns. The absence of an acrosomal reaction was characterized by fluorescence in the entire acrosome region of spermatozoa categorizing sperm cells as acrosome-intact (AI), whereas the presence of an acrosomal reaction was divided into two populations: acrosome-reacted spermatozoa with marked equatorial segment (AREQ) and complete acrosome-reacted spermatozoa (CAR).

Considering only the acrosomal reaction results, the HA selection after one-hour capacitation revealed significant differences between mature and immature sperm (MS1 14.97% vs. IS1 41.14%; *p* < 0.001).

On the other hand, significative differences were also observed between the percentage of spontaneously reacted sperm and the percentage of reacted sperm after in vitro acrosome reaction induction (NCS 10.72% vs. ARS1 41.42%; *p* < 0.001).

### 3.3. Distribution of IZUMO1 in Noncapacitated Sperm, One-Hour-Capacitated Sperm, and Selected Human Sperm by Hyaluronic Acid Test

**Figure 2:**
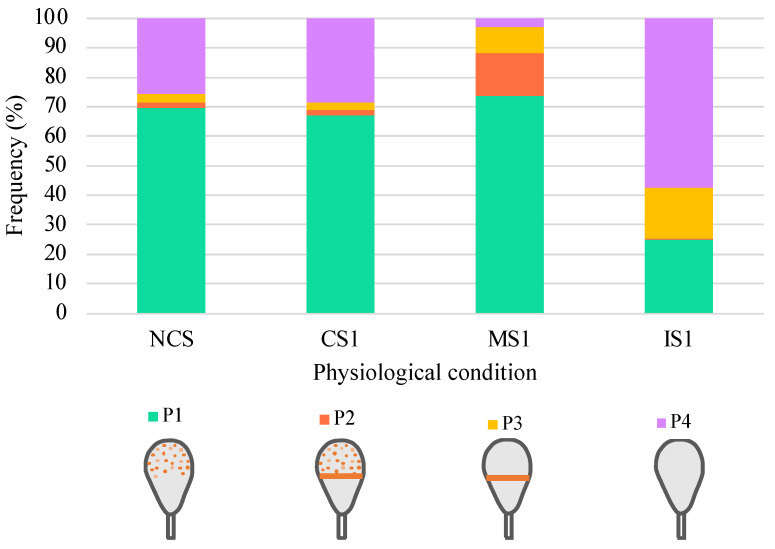
Percentages of IZUMO1 staining patterns in each physiological condition. P1, dotted fluorescence in the acrosomal region; P2, dotted fluorescence in the acrosomal region with a labelled equatorial region; P3, equatorial region labelled; P4, not labelled; NCS, noncapacitated sperm; CS1, one-hour-capacitated sperm; MS1 and IS1, mature and immature sperm selected by hyaluronic acid after one-hour capacitation.

IZUMO1 localization was classified into four different staining patterns: pattern 1 (P1) showed dotted fluorescence in the acrosomal region, pattern 2 (P2) displayed dotted fluorescence in the acrosomal region with a labelled equatorial region, pattern 3 (P3) equatorial region labelled, and pattern 4 (P4) without labeling (see staining patterns in Figure 2). Moreover, other staining patterns were observed with the post-acrosome region labelled and the homogenous head labelled. However, these distribution patterns were discarded for representing less than 5%.

Regarding the localization of IZUMO1 in each physiological condition, in the NCS condition, we recorded a high prevalence of sperm that presented P1 (~69%) and P4 (~26%) and a lower frequency of the P2 (~2%) and P3 (~3%) staining patterns. In addition, no significant differences in IZUMO1 localization were reported between NCS and CS1 (Figure 2).

Otherwise, the data obtained after selecting mature sperm by the HA test showed a high prevalence of P1 (73.47%) and a significant increase in P2 (CS1 1.81% vs. MS1 14.65%; *p* < 0.001); meanwhile, P4 decreased significantly (CS1 28.59% vs. MS1 3.01%; *p* < 0.001). In contrast, immature sperm after HA selection displayed a significant decrease in P1 (CS1 66.95% vs. IS1 24.99%; *p* < 0.001), whereas P3 and P4 increased significantly (CS1 2.64% vs. IS1 16.91%; *p* < 0.001 and CS1 28.59% vs. IS1 57.55%, respectively; *p* < 0.01). It should be noted that the P1, P2, and P4 staining patterns were significantly different (*p* < 0.001) between the mature and immature cells. In detail, P1 (73.47%) was the predominant distribution pattern of mature cells, whereas P4 (57.55%) was the major staining pattern of immature cells. Moreover, only mature sperm presented a P2 distribution pattern (MS1 14.65% vs. IS1 0.55%, Figure 2). The statistical data of IZUMO1 staining patterns in different physiological conditions (NCS, CS1, MS1, and IS1) are detailed in the Supplementary Material (Tables S1 and S2).

### 3.4. Distribution of IZUMO1 in Induced Acrosome Reaction from One-Hour-Capacitated Sperm According to Acrosomal Status

**Figure 3:**
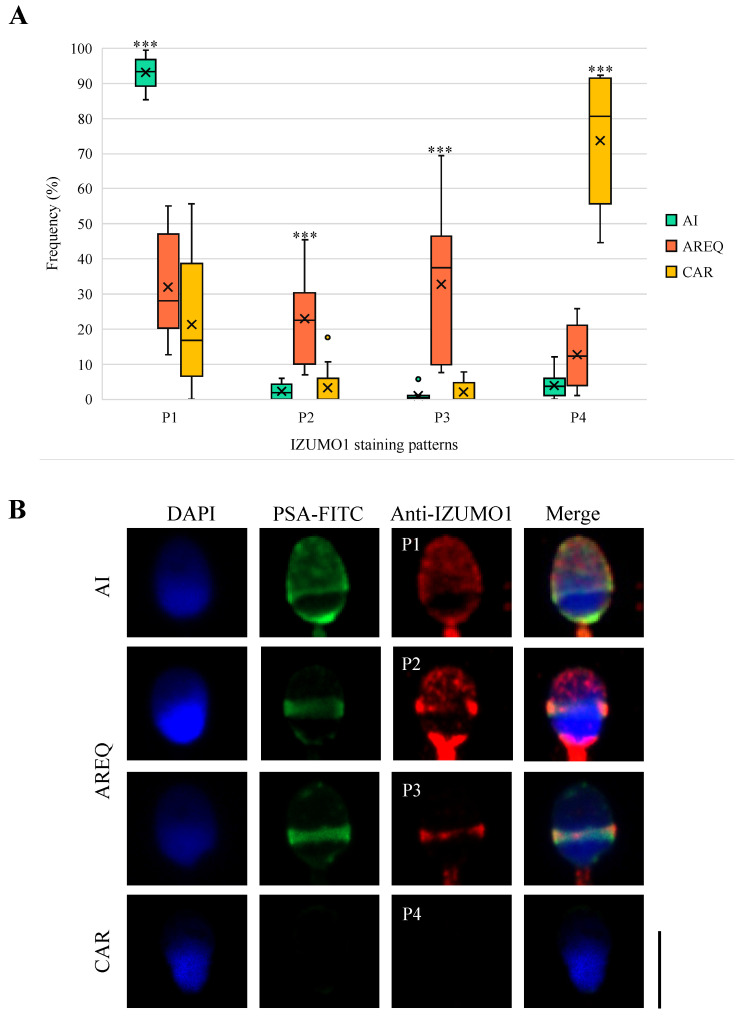
IZUMO1 immunolocation results in human sperm. (**A**) Expression and frequency of IZUMO1 staining patterns in human spermatozoa according to acrosomal status; (**B**) Costaining of IZUMO1 major distribution patterns depending on the acrosomal status of human sperm cells. AI, acrosome-intact; AREQ, acrosome-reacted with equatorial segment labelled; CAR, complete acrosome-reacted; P1, dotted fluorescence in the acrosomal region; P2, dotted fluorescence in the acrosomal region with a labelled equatorial region; P3, equatorial region labelled; P4, not labelled. Significant differences at *p* < 0.001 (***). Scale bar: 5 μm common to all images.

First, to unravel the movement of IZUMO1 after the acrosome reaction (ARS1), the results of colabeling between IZUMO1 and acrosomal status were analyzed. In terms of acrosome-intact spermatozoa (AI), 93.10% of them localized IZUMO1 significantly and mainly in a dotted staining pattern (P1; *p* < 0.001). The remaining 6.90% was distributed among the staining patterns P2 (2.20%), P3 (0.90%), and P4 (3.80%; Figure 3A).

Regarding the spermatozoa that had undergone the acrosome reaction, the following IZUMO1 staining patterns were obtained. Specifically, acrosome-reacted sperm with equatorial band labeling spermatozoa (AREQ) were significantly classified into 22.78% of P2 and 32.73% of P3 (*p* < 0.001). The remaining 35.5% was distributed between 22.70% of P1 and 12.80% of P4 (Figure 3A), whereas the results of the complete acrosome-reacted spermatozoa (CAR) show that 73.62% of them were devoid of IZUMO1 (P4, *p* < 0.001). The rest of the percentages of the IZUMO1 staining patterns were distributed in 21.28% of the cell as P1, 3.24% as P2, and only 1.86% as P3 (Figure 3A). The negative control samples did not show fluorescence, demonstrating the specificity of the procedure. Figure 3B illustrates the major and most-representative distribution patterns of IZUMO1 according to the classification of the sperm acrosome state.

## 4. Discussion

ARTs are a way of perpetuating the species. The advent of ICSI allowed infertile men to reproduce and has even been used for fertile men [33]. But how does the use of this technique affect the reproductive future of the offspring? Belva et al. [34] report a decrease in sperm count in men conceived by ICSI. This technique skips all the evolutionary methods of sperm selection, which is why PICSI has been proposed as an alternative method. The tests to evaluate the effectiveness of this new technique included the presence and distribution of different biomarkers. Previous studies of our group conducted after HA selection showed a significant increase in cells labeling HSPA2, a heat shock protein A2 involved in bringing hyaluronic acid receptors to the cell surface [29] and the requirement of the presence of SPAM1 (sperm adhesion molecule 1) throughout the sperm head to properly contact the cumulus–oocyte matrix [30]. Both studies show the localization of two proteins involved in the primary recognition of gametes; however, the location of biomarkers implicated in the next steps of fertilization is unknown. Thus, this study aims to conduct an exhaustive characterization of IZUMO1, an essential acrosomal protein involved in the adhesion and gamete fusion in human sperm cells. Particularly, our findings shed light on the distribution pattern of this protein before sperm capacitation, at one-hour capacitation, after the HA selection test, and after acrosome reaction induction.

The sperm acrosome reaction is composed of several distinct steps. Traditionally, the acrosome vesicle has been classified dichotomously as “intact” and “reacted”. However, this view is changing after different reports observed the steady state of acrosome reaction transitions [35,36,37,38]. Therefore, our investigation group considered as acrosome-intact sperm cells those cells presenting homogenous or dotted labeling in the acrosomal region, while those that presented fluorescence in the equatorial band or without labeling due to the complete loss of the acrosome content were classified as reacted spermatozoa (see Figure 3B).

The integrity of the acrosome is necessary to expose receptors, such as SPAM1, to pass through the COC [30]. Our study demonstrates a higher prevalence of acrosome integrity in mature sperm cells compared to immature sperm cells. Approximately 15% of mature sperm cells showed signs of acrosome reaction, whereas 41% of immature sperm cells exhibited acrosomal release. The acrosome reaction is a critical step for spermatozoa to penetrate the zona pellucida and establish adhesion with the oocyte. It involves the release and relocation of acrosomal proteins, allowing the sperm to expose the necessary fusion proteins and facilitate gamete interaction [39]. Our findings align with previous research indicating that after sperm capacitation, mature spermatozoa bound to HA are more likely to maintain acrosome integrity [40,41] and suggest their enhanced capability for successful fertilization.

Concerning the presence or absence of IZUMO1, we found two subpopulations of spermatozoa in noncapacitated sperm. The predominant subpopulation had IZUMO1 distributed among the staining patterns P1, P2, and P3. The remaining 26% of the population did not present IZUMO1. These results contrast with previous descriptions of IZUMO1 in bulls in which less than 5% of sperm were devoid of IZUMO1 [42]. This variation between mammalians could be because ejaculates are composed of subpopulations of spermatozoa with different characteristics; hence one cell may have a higher fertilization potential [43].

IZUMO1 staining patterns observed in human spermatozoa have been identified in other species, such as the mouse, with IZUMO1’s distribution in the acrosomal cap area, spread to the entire head and in the equatorial segment [44]. However, in bull spermatozoa, in addition to observing our staining patterns P1 and P3, another IZUMO1 distribution has been described with intense fluorescence along the border between the acrosomal principal and equatorial segments [42]. It should be noted that this distribution pattern could be the result of a different fluorescence emission due to the three dimensions of the spermatozoon. Therefore, it would reflect a variant of the equatorial region staining pattern.

Because HA is a natural sperm selector, here, we used this assay after one-hour capacitation to select and recover the subpopulation of sperm with the greatest reproductive potential. As a result of the recovery of mature sperm by using an HA test, we found that P1 persisted as a predominant distribution pattern. Interestingly, P2 was only observed in mature sperm; furthermore, a significant increase in P2 was observed in MS1 compared to CS1 (Figure 2). Therefore, as a novelty, our finding demonstrates the beginning of IZUMO1 migration (P2) in mature sperm cells to localize during adhesion in the equatorial band and, finally, fuse the gametes. According to these results, the HA selection test is an effective tool for selecting healthy sperm with intact acrosome and adequate sperm receptor distribution to carry out the fertilization process [10,29,30]. In addition, due to the law of assisted reproduction, where we cannot perform IVF in humans without reproductive purposes, this experimental design is the nearest to the physiology of the natural reproductive process.

After inducing the acrosome reaction, a costaining of anti-IZUMO1 antibody and PSA lectin was performed. Interestingly, we reported a variety of combinations between IZUMO1 staining patterns and acrosomal stages (Figure 3A). The representative distribution patterns with significant differences (*p* < 0.001) reflect the migration of IZUMO1 during the acrosome reaction in human spermatozoa (Figure 3A,B). Therefore, in acrosome-intact spermatozoa, IZUMO1 was mainly found as clusters in the acrosomal region. During the completion of the acrosome reaction, the IZUMO1 protein domains gradually moved to the plasma membrane of the equatorial segment, observing diffused clusters in the acrosomal region and concentrated in the equatorial region. Then, IZUMO1 reached the equatorial region completely. Finally, a complete loss of the acrosome content resulted in the nondetection of IZUMO1. As has been reported in rodents, there is a positive correlation between the final location of IZUMO1 and the completion of the acrosome reaction [21]. Hence, human IZUMO1 relocation is in agreement with Yanagimachi’s hypothetical view of the movement of IZUMO1 during mammalian acrosome reaction [20].

Several articles conducted in mammalian fertilization [21,42,45,46,47] report that IZUMO1 must be localized in the equatorial segment after acrosome reaction to mediate gamete adhesion as we observed in our results represented by P2 and P3. Otherwise, the sperm cells accumulate in the perivitelline space blocking the sperm–oocyte fusion and hence a successful fertilization [28]. Its accurate localization is essential, as it must interact with JUNO, which is located on the oocyte surface, mainly through the IZUMO1 central β-hairpin region [25]. As a result, JUNO rearranges IZUMO1 to generate the strength necessary to collapse the repulsion between the juxtaposing membranes through an unidentified receptor on the oocyte. Nonetheless, the oocyte and sperm need other proteins to fuse their membranes, such as SPACA6 [48] and CD9 [49]. Hence, the acrosome reaction may play an essential role in releasing and relocating the acrosomal proteins according to their function in the fertilization process, allowing the penetration of the zona pellucida, the sperm–oocyte binding, and, finally, the plasma membrane fusion [50].

## 5. Conclusions

In conclusion, our study demonstrates distinct distribution patterns of IZUMO1 in human sperm cells, with a prevalent dotted acrosome staining pattern in noncapacitated cells, one-hour-capacitated cells, and mature sperm cells. In addition, we describe for the first time in spermatozoa bound to HA the detection and increase of a new staining pattern (P2: dotted acrosomal region with a labelled equatorial region) related to IZUMO1 migration. These results provide evidence for the evaluation of the implementation of PICSI in the IVF laboratory. Further research in this area holds promise for improving infertility treatments as well as proposing new contraceptive strategies.

## Data Availability

Not applicable.

## Supplementary Materials

The following supporting information can be downloaded at https://www.mdpi.com/article/10.3390/biomedicines11112872/s1.
