# Supplementary material for: IZUMO1 Receptor Localization during Hyaluronic Acid Selection in Human Spermatozoa
Source: Biomedicines. 2023 Oct 24;11(11):2872. doi: 10.3390/biomedicines11112872 (PMC10669769; doi:10.3390/biomedicines11112872)
Supplement: Supplementary file 1 [file biomedicines-11-02872-s001.zip › biomedicines-2624669-supplementary.pdf]

Table S1. Statistical data of IZUMO1 distribution in NCS, CS1, MS1 and IS1. Data are expressed as mean ± SD (standard deviation).

Pattern	NCS	CS1	MS1	IS1
P1	69.51 ± 9.12	66.96 ± 7.19	73.47 ± 8.74	24.99 ± 21.98
P2	1.79 ± 1.28	1.81 ± 2.58	14.65 ± 5.79	0.55 ± 1.23
P3	2.90 ± 2.64	2.64 ± 2.08	8.87 ± 6.40	16.91 ± 5.57
P4	25.79 ± 7.82	28.59 ± 5.60	3.02 ± 5.22	57.55 ± 17.18

P1, showed dotted fluorescence in the acrosomal region; P2, displayed dotted fluorescence in the acrosomal region with a labelled equatorial region; P3, equatorial region labelled; P4, not labelled; NCS, noncapacitated sperm; CS1, one-hour capacitated sperm, MS1 and IS1, mature and immature sperm selected by hyaluronic acid after one-hour capacitation.

Table S2. Statistical differences of IZUMO1 staining patterns between different sperm physiological conditions (NCS, CS1, MS1 and IS1).

**Significance between Physiological Conditions**				Patterns	Physiological Condition
NCS	CS1	MS1	IS1		
-	n.s.	n.s.	***	P1	NCS
-	n.s.	***	n.s.	P2	
-	n.s.	n.s.	***	P3	
-	n.s.	*	***	P4	
	-	n.s.	***	P1	CS1
	-	***	n.s.	P2	
	-	n.s.	***	P3	
	-	**	**	P4	
		-	***	P1	MS1
		-	***	P2	
		-	n.s.	P3	
		-	***	P4	
			-	P1	IS1
			-	P2	
			-	P3	
			-	P4	

P1, showed dotted fluorescence in the acrosomal region; P2, displayed dotted fluorescence in the acrosomal region with a labelled equatorial region; P3, equatorial region labelled; P4, not labelled; NCS, noncapacitated sperm; CS1, one-hour capacitated sperm, MS1 and IS1, mature and immature sperm selected by hyaluronic acid after one-hour capacitation.
*** Bonferroni post hoc test p-value ≤ 0.001; ** Bonferroni post hoc test p-value ≤ 0.01; * Bonferroni post hoc test p-value ≤ 0.05; n.s. not significant.
